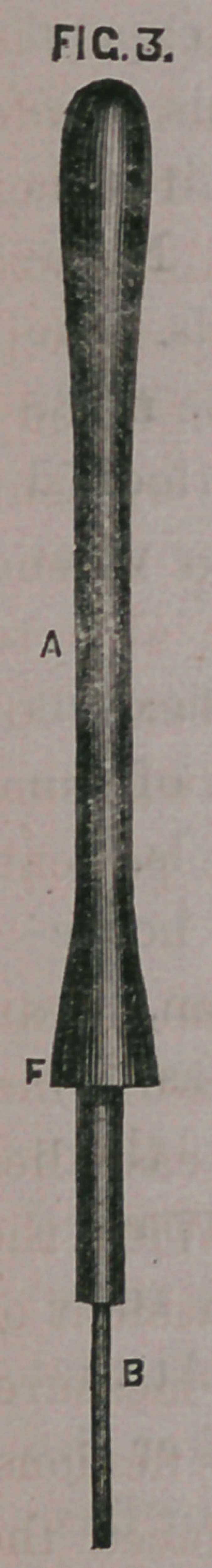# The Treatment of Uterine Flexions*Read before the Medical Association of Central New York, at Syracuse, Dec., 16th, 1873.

**Published:** 1874-04

**Authors:** Ely Van De Warker

**Affiliations:** Syracuse, N. Y.


BUFFALO

### Medical and Surgical Journal.

VOL XIII. APRIL, 1874. No. 9.

### Original Communications.

:o:

ART. I.—*The Treatment of Uterine Flexions.** By ELY VAN DE WARKER, M. D., Syracuse, N. Y.

The correction of a flexed womb by the introduction of a straight stem into its cavity is a most natural idea. Correct as it is in theory however, there has been that about it which has led to its abandonment by eminent men.

As I have before stated,† the ill success of the older form of intra-uterine stem instruments exists in an error of construction. The stem was kept *in situ* by either a (1) fixed support external to the body, or (2) by a support from the vagina. The first of these methods violates a well known physiological law. This is the law of the normal mobility of the uterus. This change in the position of the organ is incident to changes of inclination of the body, to displacing forces exerted by the vesical or rectal contents, and to pressure from above, and expulsive efforts natural to the viscera of the pelvis.‡ It is evident that in this first form of intra-uterine stem the uterus was changing, or endeavoring to change its position upon a rigid stem, which was exerting a dangerous degree of force upon the fundus, or creating great irritation of the lateral walls of the organ.

* Read before the Medical Association of Central New York, at Syracuse, Dec., 16th, 1873.
† New York Medical Journal, vol. xviii, p. 361.
‡ The Physiological Changes in the Position of the Healthy Unimpregnated Uterus. By John Williams, M. D., M. R. C. P. London Lancet; 1873, p. 593. American Edition.

The form of stem retained in position by vaginal support is not so bad. A mistake has been made in this also. This lies in the very vague idea of how fixed a body in the grasp of the vagina may be. It has therefore been thought necessary to have a large pessary for the stem to rest upon, in order to counteract its tendency to expulsion. In one of the best forms, that of Graily Hewitt, he recommends numbers 2 and 3 of his rings, embracing in the series diameters of 2¼ to 4½ inches. The numbers referred to would be 2½ to 3 inches in diameter, bending the ring into an oval, and consequently greatly increasing one diameter.* This is altogether too large for the purpose named. I have retained a stem in position for months in a woman, the mother of eleven children, by a circular vaginal flange one fourth the size named by Dr. Hewitt. He allows the stem "to fit loosely in the collar below," so as to allow "sufficient motion in *all* directions." It is evident from this quotation, that "all directions do not include ascent or decent of the organ impaled upon the stem. Neither could these movements of the uterus occur with the smallest size vaginal attachment named by him. In other words, the constricting force of the vaginal muscles would so act upon a foreign body of the size given, that its position would be firmly fixed without any reference to the constant tendencies of the womb to ascend or descend in the pelvic cavity. If a woman was ever able to wear such an intra-uterine and vaginal appliance for a series of months—as is necessary in the treatment of flexions—it was simply a case of extraordinary endurance on the part of the patient, nothing more.

The idea of support from the vagina for the retention of an intra-uterine stem is as correct in theory as is that of intra-uterine means to restore a flexion. The error, as in the former case, lies in the manner in which the theory has been utilized. With the law of normal uterine movements clearly in view, the treatment of flexions with vaginal support is a practical and valuable measure.

My first effort to divise an instrument to correct uterine flexions, consisted of a modification of Dr. Hewitt's. I first reduced the diameter of the stem at least one-half. Thus reduced, its vaginal

* Hewitt, Disease of Women, 1st. Amer. Ed. pp. 46[illegible], 522.

end was too small to admit a perforation for the end of a sound, and for this purpose I used a No. unannealed iron wire. The most important modification consisted in substituting for the enormous vaginal collar described by the author, a small adjustable vaginal flange of the least possible dimensions capable of answering its purpose. Theoretically, it should be of just sufficient size to afford grasp to the vagina to retain both stem and flange in position, and yet accommodate the changing positions of the womb. I found that the flange could be made five-eighth to three-fourths of an inch in diameter, and when of this size, could be worn and perfectly retained for months. That I had, in a measure, answered the theoretical requirements, was shown by the fact that its presence afforded exemption from pain, and, of itself never became a source of irritation. Its mode of operation it simple. The stem *a.* fig. 1, upon the end of the wire *b*, being in position, the flange, *a*, fig. 2, by means of its central perforation *c*, is placed upon the wire. Two slots *e*, upon opposite sides of the flange receive the wire, so that on being pushed up, it presents itself at the ostium vaginæ by its edge, entering the parts without difficulty, and is gently pushed upon the stem until it meets the collar *f*.

FIG. 3.

FIG. 2

FIG. 1.

Desiring to do any with the vaginal portion of the instrument,

I devised a more simple mechanism, consisting of a stem *a*, fig. 3, with a cross piece *b*, of rubber .tubing, which expanding in the cavity of the womb, acted as a means of self retention. As this has been fully described, and is here figured,* I shall give no space to its description.

Dr. Thomas Savage, of Birmingham, who fully endorses the treatment of uterine flexions by intra-uterine support, says † that any form of stem has an unaccountable tendency to slip out of the uterine cavity. I have also noticed this tendency, even after a stem had been worn—in one instance nine weeks—it was suddenly found free in the vagina. This will happen with the best of instruments. I have in a measure corrected this tendency in the self-retaining stem by carefully observing each case and placing the cross section of tubing at different points on the shaft of the stem as the case demanded, so that the rubber would expand just after clearing the internal stricture of the neck. This careful study of each case and modifying the instrument to suit it, is the surest way of preventing the expulsion of the stem. Nothing can replace this mechanical adaptation of means to ends. The self-retaining, or any other form of stem, must therefore be made to meet the special indications in each case. If this is overlooked annoyance and failure will beset the physicians, no matter what instrument he uses.

I shall illustrate the results of the treatment of uterine flexions by the intra-uterine stem, with the manner of meeting some of the difficulties which obtrude themselves, by a few clinical details.

Case I.—Mrs. L., aet. 28, native of England; occupation housekeeping. Eight years married. Mother of two children, the youngest two years old. No history of miscarriages. Was formerly a visitor to the Syracuse Free Dispensary, and was there treated for endometritis. It was also noticed that a sound passed with its curve backwards. The fundus could be felt in the retro-vaginal *cul de sac*, but well up and but slightly tender. At that time this partial flexions was not treated. Four months after she presented herself again and a most marked state of posterior flexion was found. The fundus was very sensitive to the touch, and

* New York Med. Jour., Oct, 1873; and New York Med. Rec., Dec. 15th, 1873.

† Obstet. Jour. Gr. Brit. and Ire., Nov. 1873, p. 505.

the passage of the sound gave great pain. An attempt was made to pass the stem with the vaginal flange attachment, but it was found that the womb so quickly returned to its dislocated condition that it could not at that time be introduced. The womb was thrown into position by the sound, a small weight attached to the handle of the instrument so that it would not turn and was then dropped between the thighs. The organ was thus kept in position about fifteen minutes and she was directed to return the next day.

Jan., 15th, 16th and 17th, she was given about the same length of time upon the chair. At the last date it was found that several minutes passed before the womb was completely flexed. Coughing or any movement of the body on the chair, hastened the returning dislocation. The sensibility of the cavity of the organ to the sound was also much less. The stem now passed with ease. The presence of the stem made the woman very comfortable.

Jan., 30th.—Mrs. L. returned, the stem having suddenly slipped out. Replaced the stem. Was worn almost constantly until March 7th, when the self-retaining stem was introduced. Menstruation during the wearing of the stem so far was much less in quantity, and attended with less pain.

April 5th.—Self-retaining stem worn since last date, and found well in position. The instrument removed and the patient sent home without it, in order to test the results of the treatment.

April 8th.—An examination revealed the unpleasant fact that the flexion was yet uncured. Stem again introduced.

May 20th.—Stem has been worn continuously since the 8th ult. The instrument was again removed and the patient sent home.

May 30th.—Patient presented herself for final examination. The parts were in the normal position. Discharge cured.

Nov. 15th.—Mrs. L. again presented herself with the return of all her former painful symptoms. An examination showed that the retro-flexion had returned in as marked form as before. She stated that these symptoms had been coming on for about a month. That from May 30th, until then, she had never felt better in her life. She could give no reason from exposure or over-work for the return. The self-retaining stem was introduced, since which time I have not heard from my patient.

There are two points in this case, to which I shall direct attention. The first is the preliminary restoration of the organ. By this means the stem was introduced as quickly and with as little pain as a sound would be. Without keeping the bent organ straight while the stem is being passed into its cavity, it would be a matter difficult, as well as painful to introduce the instrument. Dr. Thomas Chambers in his clinical details of many cases of uterine flexions continually refers to the difficulty of introducing the stem.* This difficulty may, in many cases, be avoided by the manipulation I have described in this case. Endometritis is usually present in cases of flexion of long standing. The stem should pass without using *any* force, if not great pain and considerable flowing will be the result of prolonged endeavors. If it is found that the stem does not easily pass, the organ ought to be again straightened, and possibly again, and again, until the stem is readily passed. This of course implies accurate curvature and great care in the use of the sound.

The other point is that a flexion, once established, has a spontaneous tendency to recur after a more or less lengthened period of restoration to a normal position. I state this that the physician may be on his guard and not too hastily pronounce his patient cured.

Case II.—Mrs. G., aet. 27, English, mother of eleven children, youngest three years old. Has always been a hard working woman. For a year previous to my seeing her had suffered from backache, bearing down, pain in defecation, tenderness over the hypogastrium, inability to walk. I found her in bed. The most painful feature of the case came from the marked nervous disturbance; sinkings, a feeling of impending death. Sleep difficult, appetite gone, and general health much impaired. An examination showed an acutely retro-flexed womb, os patulous with lateral rents. The sound penetrated $3\frac{1}{4}$ inches, and cavity of the organ was tender and disposed to bleed. The flexed organ was easily returned to its normal position but quickly recoiled to its dislocated position on the withdrawal of the sound. For several weeks the only treatment given was to the general health.

* Obstet. Journal, Gr. Brit. and Ire. Vol. 1, five Nos.

June 20th.—Introduced the flanged stem.

June 24th.—The stem is well borne, the pain and hypogastric tenderness abating.

July 13th.—The patient is now going about the house attending to her domestic duties. She is sleeping well, appetite good, gaining flesh and strength. The nervous attacks are less frequent and severe.

July 30th.—The patient at this visit complained of pain and difficulty in passing water; an examination found the womb strongly retro-versed, the flange pressing the neck of the bladder. The womb was put into its proper position and the posterior vaginal *cul de sac* tamped with carbolized cotton wool, forming a broad, soft bed for the organ to rest upon.

Nov. 10th.—Since the last date the stem has twice escaped, remaining out on one occasion ten days. Without the support of the stem the uterus gradually returns to its flexed position. The general health of the patient is good, and she is leading a useful life.

When this patient will be cured of the flexion I shall not venture to predict. All that I can claim for the treatment is, that this woman has been taken from her bed and made useful to herself and other. A positive cure after wearing the stem for six months almost continuously, seems as distant now, as when the treatment was commenced.

The correction of the retroversion in this case is a point of interest. There is, I have noticed, a tendency to retroversion after the correction of a retro-flexed uterus by means of the intra-uterine stem. The flexion removed by the presence of the stem, the fundus of the organ has a disposition to at once drop backward into the Douglas space. To correct the double tendency to displacement by a vaginal appliance connected with the stem, is precluded by my theory of the intra-uterine stem. The office of the stem is to correct a flexion, and any other indication is to be met independently of the stem or its necessary attachments. The best means I have found to prevent the retroversion is—as in this case—to fill the posterior *cul de sac* of the vagina by a mass of cotton wool, saturated with a solution of carbolic acid in glycerine. It is efficient, painless and cleanly. The tamping may be removed every ten days. The mass of cotton must be just sufficient to fill the space and not project beyond the neck into the grasp of the vagina or it will be forced out with the stem.

Case III.—Mrs. H. act. 29, widow, the mother of one child six years old. Has been a widow four years. For the last three years menstruation has been very abundant, often extending over two weeks, and several times there has been a continual discharge of color for two and three months. Latterly there has been a sore and painful point in the right iliac region, backache and bearing down. An examination revealed an open and everted os, a marked backward flexion with right lateral inclination. The sound penetrated three and one-quarter inches, and was attended with acute pain, and nearly twenty-four hours passed before the pain of the examination ceased. The depressed fundus was also exquisitely sensitive to the touch. The general health of the woman was greatly impaired, and her resolution and "pluck" completely destroyed. As it would be impossible to treat the flexion in the present state of the parts, a course of preliminary treatment was undertaken. Suppositories of morphia and belladonna, vaginal irrigation and the intra-uterine caustic instrument, properly curved, was carefully but thoroughly used.

March 8th.—For the second time the cavity of the uterus was cauterized. Considerable pain followed, which was met by morphia hypodermically.

April 3d.—The curved probe can be passed without exciting a great degree of pain, and I thought this day I would venture the introduction of the stem. The flange instrument was used. Although the flexed organ quickly recoiled when the probe was removed, I introduced the stem readily in the following manner; the probe in position, the stem was passed along side of it as far as possible into the neck, as the probe was withdrawn, the stem was pushed forward, and thus the stem was passed into the cavity before the body of the uterus had time to recoil to its flexed position.

April 9th.—The stem is well borne. The vaginal douche ordered continued.

May 4th.—The last menstruation just passed, was more healthy in time and quantity than any in the last three years. The sore point in the right iliac region has disappeared; she is now able to take long walks and has a better color. She complains of pain and some soreness of the pubic space. A turpentine ointment was ordered as an application to this point.

May 10th.—The self-retaining stem was introduced but it slipped out after the woman reached home. Thinking that the cross section of rubber tubing was too near the end of the stem, and also that the internal stricture of the neck was remarkably open, a larger piece of tubing was passed through the stem nearly an inch from the end, and on May 15th, the instrument was again introduced.

July 8th.—Came to me with the stem out, the stem had made its escape several days before, it had also been expelled on two other occasions previous to this date. An examination proved that the uterus was in its normal position, the os slightly enlarged, the neck firm and smooth, no tenderness when the body of the organ was pressed. The sound penetrated three inches and elicited but a slight degree of tenderness. Menstruation slightly more abundant than in health at times, otherwise normal.

This is the last examination made in this case. I have seen the lady several time, and have heard no complaints. The result in this interesting case—so far as the treatment of flexions is concerned, is anomalous. A most potent course of treatment, other than the use of the stem was followed, which may in a great measure account for the rapidly good effects. Endometritis, I believe, to be a natural complication of flexions. What may be the relation of cause and effect in this conjunction of the two diseases I am not prepared to say. If however the latter is the result of the first, rational medicine at once suggests the employment of means to arrest the course of the disease in the cavity, conjointly with the mechanical reduction of the dislocation of the organ. The application of the nitrate of silver was not in this case applied with any such intention. We all know how rapidly the morbid sensibility of a diseased surface is removed by a free use of the caustic, and it was for the purpose of blunting the exquisite tenderness of the lining membrane that I employed it in this case. The freedom from pain after the introduction of the stem proved its value. The first passage of the sound is often more painful than any subsequent use of the instrument. In the natural history of the uterus there is no point of more interest than the readiness with which it accommodates itself to changes and to intrusions into its cavity. By frequent use of the sound the morbid sensibility may be greatly modified. In one case—and as this is the only point of interest it is not worth while to detail—the frequent employment of the sound, covering a period of about three weeks, mitigated the tenderness of the lining membrane greatly.

Apropos to this, Dr. Savage* makes a strange statement "that it (the stem) is more likely to be retained in the endometritic uterus, in consequence of the abnormal and *insensittve* state of the mucus membrane." It is but justice to Dr. Savage to say, that this was "suggested" to him and not originated. Comment is not necessary upon this, as it violates all our experience, and I referred to it here because it was allowed place in the paper of such an able man.

During the wearing of the stem, the diligent use of the vaginal douche is an important matter. If the stem has been in position several weeks semisolid, whitish deposits are apt to accumulate around the vaginal portion of the instrument which may become a source of irritation. This the current of water will wash away. As a depletory measure it is potent, and the simplest within our reach. Half a gallon of tepid water night and morning, flowing from a fountain syringe, is about the quantity to use.

Case III.—Mrs. B. of Homer, N. Y., American, aet. 44, married 29 years. Mother of four children, age of oldest 24, that of the youngest 15 years. Had two miscarriages. Some ten years ago had a fall, since which time she has suffered from a uterine trouble and gradual failing of the general health. Later symptoms may be said to involve the nervous system more than the reproductive organs. An examination showed the os to be directed slightly forward, the cervix large and softened with the fundus uteri distinctly felt posteriorly. The sound verified the diagnosis of acute backward flexion of the uterus.

* Loc. sit., p. 505.

April 12th.—I was called to Homer in consultation with Dr. C. Green. We both attributed Mrs. Bs'. condition of nervous prostration to the uterine deformity, and we both attempted to introduce a variety of intra-uterine stem instrument known in the catalogues as the Edward's pessary, a most extraordinary and worthless affair. We failed to introduce the instrument. The reasons of failure were on account of the large size of the vaginal portion, and the great size of the stem, being over three-eights of an inch in diameter. She was placed upon a course of tonic and sedative treatment, followed by slight amendment. In the early part of July she went upon a visit near the sea-side, and returned in September. During this period she had been without treatment of any kind. The effect of the change of air and scene, and rest, was magical. She had in this short time gained flesh, and the tone of the nervous system was almost completely restored.

September 6th.—An examination revealed the same state of uterine flexion existing. The self-retaining stem was introduced at my office, and the lady took the cars for her home. Two days after, I received a letter from her stating that she had considerable pain and difficulty in urination, backache and difficulty in walking. These symptoms were owing to an extreme degree of retroversion. The stem was worn about a week and then removed by Dr. Green.

No further treatment given.

Now, a patient who is being treated by the intra-uterine stem ought to be under observation, or if obliged to leave she should be attended by her family physician. Had Dr. Green seen this case earlier I am sure the stem could have been retained—so liable is the uterus to assume a position of retroversion after the correction of a flexion (retro) that this alone would be a sufficient reason for keeping the patient under observation. A slight degree of version in no way complicates the wearing of the stem, but any sudden exertion may convert this into a more advanced form of the dislocation, and which would demand correction.

So important do I regard care and gentleness in the manipulation necessary in the treatment of flexions, that I shall make a suggestion or two in regard to the introduction of the stem. In addition to the measures already detailed I found considerable advantage in some cases of filling the posterior *cul de sac* of the vagina with cotton after the womb was thrown into position, and with the sound still *in situ.* This would keep the uterus sufficiently straight for the stem to glide easily into position. If the tendency to retroversion has been marked the pessary of cotton-wool may be left in position. In this case however, care must be taken to have the pessary of proper size, otherwise if too large, it will be forced out of place and lead to the expulsion of the stem.

Another plan of inserting the stem is that practiced by Dr. Thomas Chambers, of the Chelsea Hospital for women, and highly recommended by him.* Although I have never tried it, it appears to be an efficient measure. This method requires the introduction of Sim's Speculum, the neck of the uterus is then seized by the vulsullum and drawn down, while the fundus is pressed upward with the fingers; this straightens the body of the organ, and while the cervix is firmly held the stem is inserted by the other hand.

In the matter of the flexed uterus having contracted adhesions we must exercise great care. If the adhesions are firm, it is evident that the fundus could not ascend from its depressed position, and if the stem were introduced, the body of the uterus could be straightened by thowing the neck violently upward and forward in case of a retro-flexion and in the opposite direction in the reverse deformity, thus placing the organ at right angles to the track of the vagina. Such a state of affairs would result in a worse condition than that of an uncorrected flexion.

In a case now under treatment, in which I have finally introduced the self-retaining stem with comfort to the woman, I could not throw the uterus in position by the sound on account of the great pain caused by attempts to do this; but, at the expense of much time, I succeeded in forcing the fundus upward and forward with a pair of sponge probangs after the manner of Dr. Sims† in retroversion. By this manipulation, often repeated, the fundus

* Obstet. Journal, Gr. Brit. and Ire. Vol. I, page 322.

† Clinical Notes on Uterine Surgery; page 253.

yielded more and more readily from its retro-flexed position by either the breaking up of the adhesions, or by an elongation of the fibres of adventitious tissue. If the adhesions resist this method, I cannot suggest any means of overcoming them. In a case of moderately firmly abnormal union between the parts, a combination of the method of Sims in retroversion, and of Chambers in flexion, might be used to great advantage. However, it is not so much a question of ease in the introduction of an intra-uterine stem in these cases as it is to bring the uterus into a state favorable to the wearing of a stem. We can not trust an intra-uterine stem to break up, or relax old adhesions. This must be a matter of preliminary treatment. In treating these adhesions we must recollect that they are traces of parametritic inflammation, that they may not be traces alone, but inflammation may be lurking potentially in the tissues of the parts, and rough or untimely handling may rouse it into dangerous activity.

While I have written this paper in advocacy of the use of the intra-uterine stem, not only as a means of cure, but for what is of almost equal importance, as a measure of releif from present pain; still I feel bound to urge judgment and care in its employment.

NOTE.—For the information of those who may wish to test the value of these instruments I will state that they may be had of Messrs. Shepard & Dudley, 150 Willliam Street, New York.

## Figures and Tables

**FIG. 1. f1:**
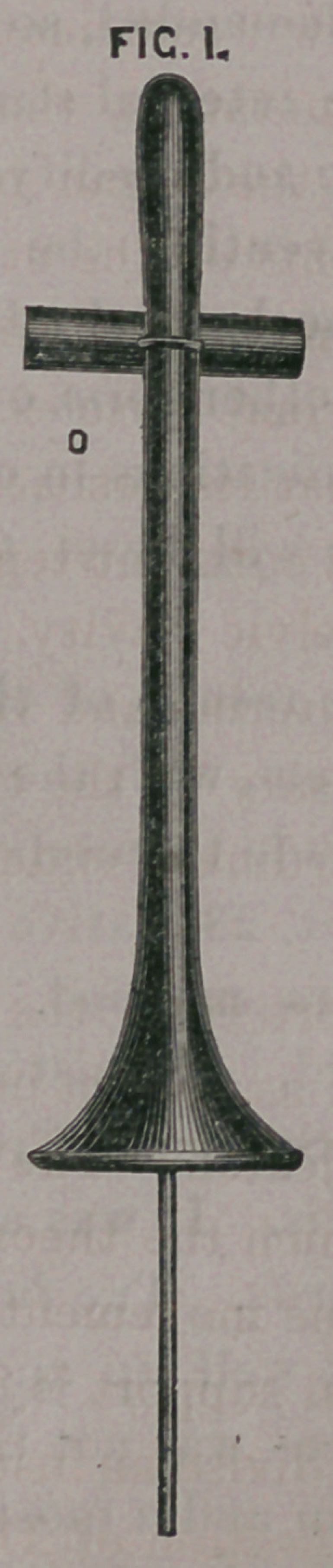


**FIG.2 f2:**
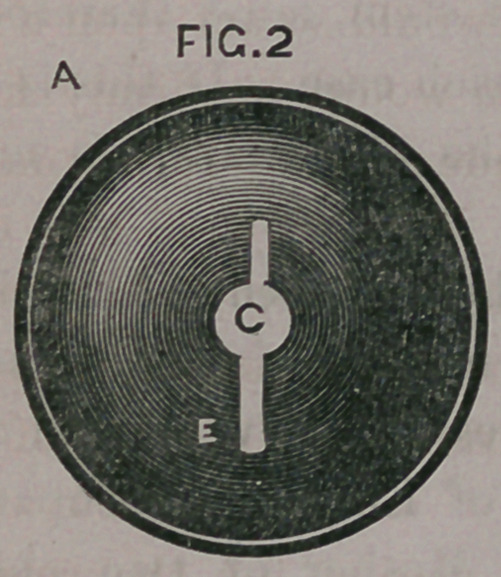


**FIG.3. f3:**